# Application of Artificial Intelligence in the Analysis of Features Affecting Cataract Surgery Complications in a Teaching Hospital

**DOI:** 10.3389/fmed.2020.607870

**Published:** 2020-12-11

**Authors:** Michele Lanza, Robert Koprowski, Rosa Boccia, Katarzyna Krysik, Sandro Sbordone, Antonio Tartaglione, Adriano Ruggiero, Francesca Simonelli

**Affiliations:** ^1^Multidisciplinary Department of Medical, Surgical and Dental Specialities, University of Campania Luigi Vanvitelli, Naples, Italy; ^2^Institute of Biomedical Engineering, Faculty of Science and Technology, University of Silesia in Katowice, Sosnowiec, Poland; ^3^Department of Ophthalmology with Pediatric Unit, St. Barbara Hospital, Trauma Center, Sosnowiec, Poland

**Keywords:** complications, artificial intelligence, time of surgery, risk factors, cataract surgery

## Abstract

**Aims:** To evaluate the ocular and systemic factors involved in cataract surgery complications in a teaching hospital using artificial intelligence.

**Methods:** One eye of 1,229 patients with a mean age of 70.2 ± 10.3 years old that underwent cataract surgery was selected for this study. Ocular and systemic details of the patients were recorded and then analyzed by means of artificial intelligence. A total of 1.25 billion simulations of artificial intelligence learning and testing were conducted on several variables and a customized model of analysis was developed.

**Results:** A total of 73 complications were recorded in this study. According to the analysis performed, the main factors involved in cataract surgery complications were: a surgeon in training, axial length and intraocular lens power. The model predicted how long surgery would last with an error of <6 min compared to the effective time needed.

**Conclusions:** According to the data here obtained, artificial intelligence could be an interesting option to build customized models able to prevent complications and to predict actual surgery time. The customized algorithm option allows the development of better models adaptable to different units as well as the possibility to be calibrated for the same unit along time.

## Introduction

Cataract, the opacification of the human eye lens, leads to a reduction of visual acuity and is largely, diffused among adults, affecting almost one hundred million people worldwide (1).

The only effective therapy for cataract is surgery and, currently, the standard treatment is phacoemulsification with intraocular lens (IOL) implant in the capsular bag (2, 3).

Improvements in instrumentation, technology, training programs, surgical techniques, design of intraocular lenses, and medications have led cataract surgery to become a very safe and effective procedure, able to restore visual acuity and to improve quality of life (1). Consequently, the expectations of patients shifted from the need of restoring vision, with or without spectacles, to the demand of obtaining a very good visual acuity, without spectacles, and being disappointed in case of discomfort consequent to the surgical procedure (1, 3).

Nowadays, patient expectations induce physicians to limit the complication rate as much as possible and this is obviously more difficult in a teaching hospital. Today, intraoperative and postoperative cataract surgery complications are <10% and are usually mild and transient although in a few cases these events can lead to long-term visual dysfunction with strong dissatisfaction of patients (4). Most frequent intra-operative complications are posterior capsule rupture with or without vitreous loss (0.5–5.2%), intraoperative iris floppy syndrome or iris prolapse (0.5–2%) and iris or ciliary body injury (0.6–1.2%) (1).

For this reason, many studies have evaluated the most frequent features involved in cataract surgery results (4–14).

However, prediction on surgery time is a topic that has not been covered in the most important studies published on risk stratification in cataract surgery, there are few reports suggesting a mean time of 22.1 min for every procedure (10). The surgical time could be influenced by many variables such as the kind of cataract, the collaboration of the patient, concomitant risk factors (both ocular and systemic ones) and the experience of the surgeon (10).

Artificial intelligence (AI) enables to acquire information about a specific working situation and obtain strategies to improve the quality of that work. AI application in medicine and ophthalmology started some years ago (15–22) and its use is increasing (23–25) but it has never been applied to this specific area.

The purpose of this study is to evaluate the incidence of complications occurring during cataract surgery in a real life scenario of a teaching hospital with AI and to use these data to build a model to detect risk factors for intraoperative complications and to predict surgery time, in order to optimize the outcomes also when residents are performing the surgery.

## Materials and Methods

This retrospective study evaluated the charts of patients undergoing cataract surgery from January 2018 to December 2019. A total of 1,229 eyes of 1,229 patients were collected. General and clinical characteristics of the population study are summarized in Tables 1, 2.

**Table 1:** Means, standard deviations (SD) and ranges of numerical parameters included in the study.

	**Mean ± SD**	**Range**
Age (year)	70.2 ± 10.3	10 to 94
BCVA (snellen lines)	0.27 ± 0.19	0 to 0.95
Sphere (D)	−1.36 ± 4.27	−30 to +8.5
Cylinder (D)	−0.18 ± 0.95	−5 to +4.5
Spherical equivalent (D)	−1.45 ± 4.45	−30 to 8.75
IOP (mmHg)	15.25 ± 2.21	8 to 24
Axial length (mm)	23.89 ± 2.08	17 to 35.82
Mean Keratometry (D)	44.16 ± 1.74	33.24 to 52.12
IOL power planned to implant (D)	+20.15 ± 5.59	−7 to 34
Endothelial cell count	2,410.59 ± 294.99	1,293 to 3,213
ACD (mm)	3.09 ± 0.48	2.23 to 4.03
Surgery time (minutes)	17.58 ± 9.42	5 to 85

*BCVA, Best correct visual acuity; IOP, intraocular pressure; IOL, intraocular lens; ACD, anterior chamber depth*.

**Table 2:** Characteristics of patients included in the study and surgical procedures.

	**Male**		**Female**	
Sex	557 (45%)		672 (55%)	
	Right		Left	
Eye	578 (46.9%)		653 (53.1%)	
	Trained surgeon		Resident	
Procedures	1,043 (85.3%)		181 (14.7%)	
	Total	Cortico-nuclear	Sub capsular	Cortico-nuclear and sub-capsular
Cataract type	62 (5%)	765 (62.2%	76 (6.2%)	326 (26.5%)
	General	Topical	Sub tenonian	Peribulbar
Anesthesia	4 (0.3%)	203 (40.9 %)	269 (21.9 %)	453 (36.9%)

**Figure 1:**
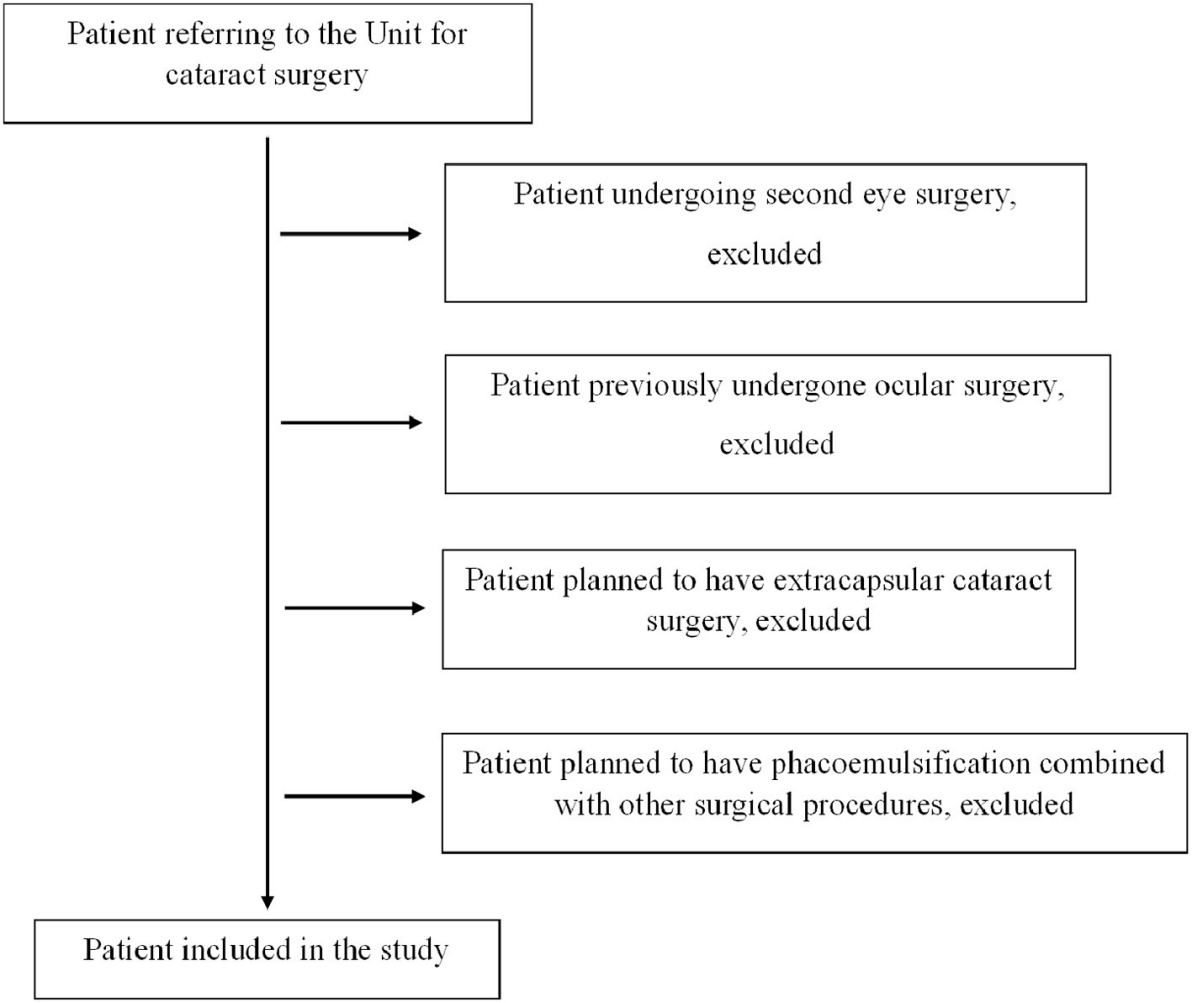
Flow chart showing the exclusion criteria for this study.

If a patient underwent surgery in both eyes, only information related to the first eye was included to limit bias, considering that a better knowledge of the patient related to first eye surgery could limit the complications during second eye phacoemulsification. This approach was chosen also to avoid bias in the statistical analysis related to the inner correlation between pair organs. The study included only eyes treated with phacoemulsification and IOL implant in the capsular bag without previous ocular surgery. Patients undergoing cataract surgery with extra-capsular technique or with phacoemulsification and IOL implant combined with other surgical procedures such as trabeculectomy, vitrectomy or corneal transplant were excluded. Patients affected by other ocular diseases such as glaucoma, diabetic retinopathy or macular degeneration were included if they had no previous ocular surgery (Figure 1). All patients underwent an anesthesiologic visit and a complete eye examination with retinal OCT scan and IOL Master evaluation before surgery. The list of the ocular and systemic diseases affecting the population study is represented in Table 3.

**Table 3:** List of concomitant diseases.

**Ocular diseases**	**Number**	**%**
None	681	60.3%
Glaucoma	122	10.8%
PEX	18	1.6%
DR	11	1.0%
PDR	52	4.6%
Amblyopia	19	1.7%
Maculopathy	138	12.2%
Corneal dystrophy or degeneration	80	7.1%
Trauma or congenital malformations	19	1.7%
Previous retinal tears treated with argon laser	5	0.4%
Corneal alteration due to previous refractive surgery	6	0.5%
**Systemic diseases**		
None	226	18.4%
BPH	15	1.3%
NIDMD	37	3.3%
Hypertension	297	26.3%
Abnormal heart conditions	27	2.4%
Respiratory problems	47	4.2%
IDMD	24	0.9%
Previous cerebral ictus	8	0.6%
Other	23	2.0%

The surgeon was either well-experienced or a 3rd or 4th year resident in ophthalmology, only these trainees were included because they had better surgical experience. In our residency program, indeed, 1st year residents have no surgical experience and 2nd year ones have only adnexa surgical experience, cataract surgery starts from the 3rd year. All the operators were right-handed, every procedure planned was phacoemulsification with IOL in the bag implant. IOL power was calculated with SRK/T formula for eyes with axial length longer that 22 mm and with Hoffer Q formula for those shorter than 22 mm, the target refraction was emmetropia or that agreed upon with the patient according to his/her needs. Constellation Vision System with Ozil Torsional phaco tip (Alcon, Fort Worth, Texas, USA) was used, with temporal approach and a 2.75 mm clear corneal incision. IOL models implanted in the population study were mostly SA60AT (647; 52.6%) and SN60WF (265; 21.5%) (Alcon, Fort Worth, Texas, USA).

The study adhered to the tenets of the Declaration of Helsinki and informed consent was obtained for both the surgical procedure and personal data processing.

The parameters included in the artificial intelligence investigation were the following: age, sex, laterality of the eye, systemic diseases, ocular diseases, cataract type (maturity classification), best correct visual acuity (BCVA), spherical defect, cylinder defect, spherical equivalent, intraocular pressure (IOP), intraocular lens (IOL) Power, axial length (AL), maximum keratometry, minimum keratometry, mean keratometry, anterior chamber depth (ACD) measured from the epithelium, endothelial cell count, anesthesia, surgery time, extra devices used during surgery, kind of surgeon (trained or in training).

Both IOL power and axial length have been included in the study because also corneal power is needed, and sometimes anterior chamber depth, to determine the IOL calculation therefore, the first two parameters can not be considered perfectly inversely proportional.

All data combinations were analyzed. This means that parameters were first introduced in the AI system and then checked. For example, anesthesia was first introduced and then checked to verify whether it could be used to divide patients with and without surgical complications. Next, whether patients could be divided with or without surgical complications based on anesthesia and cataract type. And so on for all data combinations (peer-to-peer). There were 4,194,303 such data combinations. According to the state of the art, the artificial intelligence method was based first on learning, and then it was tested to verify whether it learned correctly by measuring Accuracy (ACC), Specificity (SPC) and sensitivity (TPR). The simulations were made using a computer with an Intel Xeon 3.3 GHz processor, 12 GB RAM. The algorithm was written in the Matlab Version: R2016a, with toolboxes Statistics and Machine Learning Toolbox Version 10.2 (R2016a). Considering that the data groups with and without surgical complications must be equal, patients without complications were randomized each time. In total, 1.25 billion simulations of artificial intelligence learning and testing were conducted.

At the initial stage, the classifier was selected from the following types: neural networks, discriminant analysis, naive Bayes classifier, binary decision trees and support vector machine. This test was performed to identify which classifier brought to the best results. The selection criterion adopted was ACC being the maximum value obtained when checking the classifier performance with all possible combinations of features (peer-to-peer) for 5 different classifiers. The results obtained are shown in Table 4, illustrating that discriminant analysis (ACC to 87.2 ± 11) gave the best results.

**Table 4:** Results obtained for five different types of classifiers and standard deviation of the mean (std) for 1,000 patient randomizations.

**Classifier type/result**	**SPC (%)**	**TPR (%)**	**ACC (%)**
Neural networks	57.2 ± 8	88.5 ± 22	69.1 ± 21
Discriminant analysis	62.2 ± 22	82.3 ± 20	87.2 ± 11
Naive Bayes classifier	61.7 ± 23	87.7 ± 15	72.1 ± 9
Binary decision trees	61.0 ± 11	78.5 ± 20	63.2 ± 10
Support vector machine	59.3 ± 19	71.2 ± 13	71.2 ± 9

At the next stage, this analysis was elaborated to obtain five different types of discriminant analysis: linear, diaglinear, quadratic, diagquadratic, mahalanobis. As shown in Table 5, the best classification result was diaglinear type discriminant analysis (ACC = 87.2 ± 11) that was therefore selected for further analysis and selection of the most significant features.

**Table 5:** Results obtained for five different types of discriminant analysis and standard deviation of the mean (std) for 6,000 patient randomizations.

**Discriminant analysis type**	**SPC (%)**	**TPR (%)**	**ACC (%)**
Linear	59.4 ± 18	87.3 ± 13	67.4 ± 11
Diaglinear	62.2 ± 22	82.3 ± 20	87.2 ± 11
Quadratic	61.9 ± 12	88.2 ± 11	82.2 ± 18
Diagquadratic	58.1 ± 11	75.5 ± 13	67.4 ± 9
Mahalanobis	61.7 ± 9	72.4 ± 15	67.3 ± 15

All the mentioned analysis methods were created by the authors for the purposes of this paper.

After checking the normality of the distribution with Kolmogorov-Smirnov test, difference between actual surgical times and the predicted ones was evaluated with paired Student *T* test and with mean square error (MSE).

## Results

Analyzing the charts, 73 eyes had complications during cataract surgery. Particularly, capsular tear not requiring vitrectomy was observed 19 times (26.03%), capsular tear requiring vitrectomy 32 times (43.84%), ocular hypertension with iris prolapse in the main incision requiring intraoperative mannitol use to continue surgery occurred 13 times (17.81%), capsular tear that forced to perform unplanned extracapsular cataract extraction occurred 4 times (5.48%), capsular tear with nucleus drop in vitreal cavity occurred 4 times (5.48%), vitreous loss without capsular tear requiring anterior vitrectomy occurred once (1.37%). In 48 out of 73 cases, eyes underwent cataract surgery with a trained ophthalmologist and in 25 cases with a resident.

### Which Features Affect Surgical Complications?

First ten best results for some selected feature configurations for the highest ACC values is shown in Table 6A (for diaglinear type discriminant analysis classifier).

**Table 6:** Progression of the analysis related to detect features involved in cataract complications, **A**. top ten best results; **B**. top ten best results but without surgery time; **C**. Number of occurrences of individual features for ACC > 72%.

**A: SELECTED FEATURE CONFIGURATIONS FOR THE HIGHEST ACC VALUES (FIRST TEN BEST RESULTS)**																						
**Age**	**Sex**	**Eye**	**Systemic disease**	**Ocular disease**	**Cataract type**	**BCVA**	**SP**	**CYL**	**SE**	**IOP**	**IOL power**	**al**	**K1**	**K2**	**Average_k**	**ACD**	**Endo cell count**	**Anestesia**	**Surgery time**	**Extra device**	**Kind of surgeon**	**ACC**
0	0	0	0	0	0	0	0	0	0	0	1	1	0	0	0	0	0	0	1	0	0	87
0	0	0	0	0	0	0	0	1	0	0	0	0	0	0	1	0	0	0	1	0	0	86
0	0	0	0	0	0	0	0	1	0	0	0	0	1	0	0	0	0	0	1	0	0	86
0	0	0	0	1	0	0	0	1	0	0	0	0	0	0	0	0	0	0	1	0	0	86
0	0	0	0	0	0	0	0	0	0	0	0	0	0	1	0	0	0	0	1	0	0	85
0	0	0	0	0	0	0	0	0	0	0	0	0	1	0	0	0	0	0	1	0	0	85
0	0	0	0	0	0	0	0	1	0	0	0	0	0	0	0	0	0	0	1	0	0	85
0	0	0	0	0	0	0	0	1	0	0	0	0	0	1	0	0	0	0	1	0	0	85
0	0	0	0	0	1	0	0	0	0	0	0	0	0	0	1	0	0	0	1	0	0	85
0	0	0	0	0	0	0	0	0	0	0	0	0	0	0	1	0	0	0	1	0	0	85
**B: SELECTED FEATURE CONFIGURATIONS FOR THE LARGEST ACC VALUES FOR WHICH THERE IS NO SURGERY TIME**																						
**Age**	**Sex**	**Eye**	**Systemic disease**	**Ocular disease**	**Cataract type**	**BCVA**	**SP**	**CYL**	**SE**	**IOP**	**IOL power**	**al**	**K1**	**K2**	**Average_k**	**ACD**	**Endo cell count**	**Anestesia**	**Surgery time**	**Extra device**	**Kind of surgeon**	**ACC**
0	0	0	0	0	0	0	0	0	0	0	1	1	0	0	0	0	0	0	0	0	1	76
0	0	0	0	1	0	0	0	0	0	0	0	0	0	1	0	0	0	0	0	0	1	75
0	0	0	0	1	0	0	0	0	1	0	0	0	0	0	0	0	0	0	0	0	1	75
0	0	0	1	0	0	0	0	0	0	0	0	0	0	0	0	0	0	1	0	0	1	75
0	0	0	0	0	0	1	0	0	1	0	0	0	0	0	0	0	0	0	0	0	1	74
0	0	0	0	1	0	0	0	0	0	0	0	0	0	0	1	0	0	0	0	0	1	74
0	0	0	0	1	0	0	0	0	0	0	0	0	1	0	0	0	0	0	0	0	1	74
0	0	0	0	1	0	0	0	1	0	0	0	0	0	0	0	0	0	0	0	0	1	74
0	0	0	0	1	0	0	1	0	0	0	0	0	0	0	0	0	0	0	0	0	1	74
0	0	0	0	1	1	0	0	0	0	0	0	0	0	0	0	0	0	0	0	0	1	74
**C: NUMBER OF OCCURRENCES OF INDIVIDUAL FEATURES FOR THE ADOPTED THRESHOLD ACC > 72%**																						
**AGE**	**Sex**	**Eye**	**Systemic disease**	**Ocular disease**	**Cataract type**	**BCVA**	**SP**	**CYL**	**SE**	**IOP**	**IOL power**	**AL**	**K1**	**K2**	**Average_K**	**ACD**	**Endo cell count**	**Anestesia**	**Surgery time**	**Extra device**	**Kind of surgeon**	**ACC**
14	10	13	5	20	15	13	9	17	13	9	8	9	20	20	20	8	11	13	141	0	15	>72

*“0” - the feature is not included in the classification; “1” - the feature is included in the classification. BCVA, Best correct visual acuity; SP, sphere; CYL, cylinder; SE, spherical equivalent; IOP, intraocular pressure; IOL, intraocular lens; AL, axial length; K, keratometry; ACD, anterior chamber depth; endo, endothelial*.

**Figure 2:**
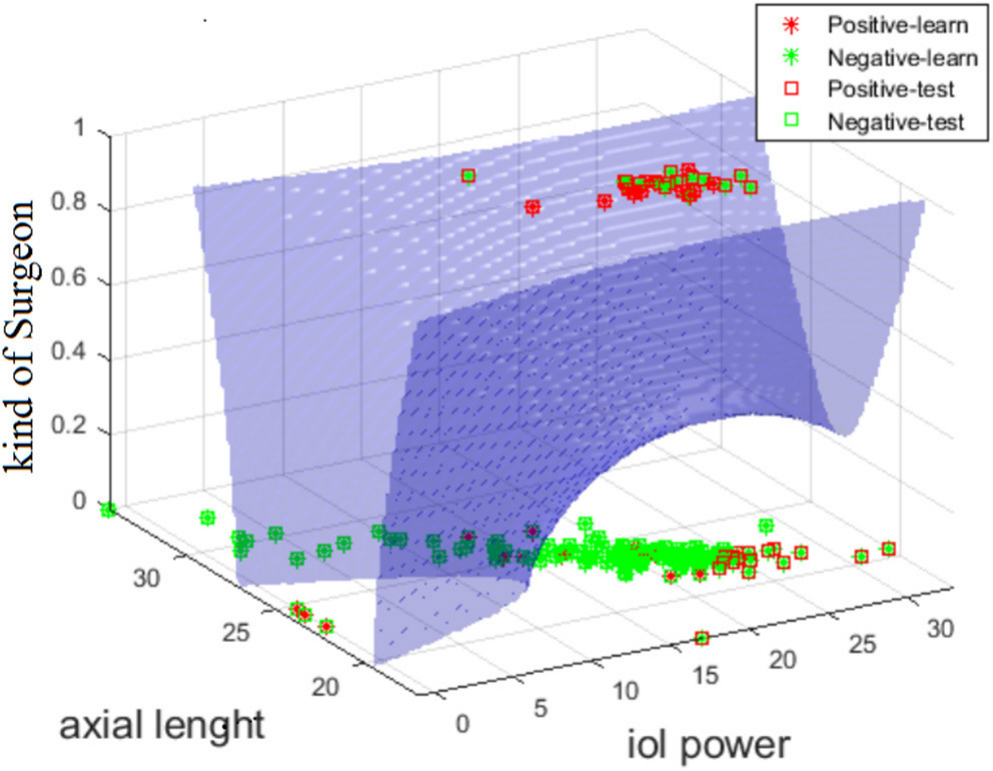
Graph showing the curve of division of cases with complications (red) and without complications (green) for the diagquadratic discriminant analysis for the features: al, iol power, typology of Surgeon.

Surgery time, in most cases, depends on the presence or absence of complications but it may also depend on individual patient features, therefore the latter appeared as an input feature. When it is removed from the input vector, ACC values decrease by approximately 10%. Some selected feature configurations for the largest ACC values for which there is no surgery time are shown in Table 6B. According to the ACC values presented in this table, the kind of surgeon is most common and ACC values range from 76 to 74%. The combination that provided strongest results was the one between “Kind of Surgeon” + “Axial Length” + “IOL Power” (Figure 2). The graph of the number of occurrences of individual features is interesting from a diagnostic point of view and illustrates the practical use of the presented analysis. Since the number of occurrences of each of the 22 features range from 0 to 141 times, a restriction has been introduced. This limitation is the adopted ACC value, which is 72%. The table with the number of occurrences of individual features is shown below (Table 6C). The most common feature among those present in the training and test vector is surgery time occurring 141 times, and the least common are systemic disease 5 times and extra device 0 times.

### Is It Possible to Predict Surgery Time?

**Figure 3:**
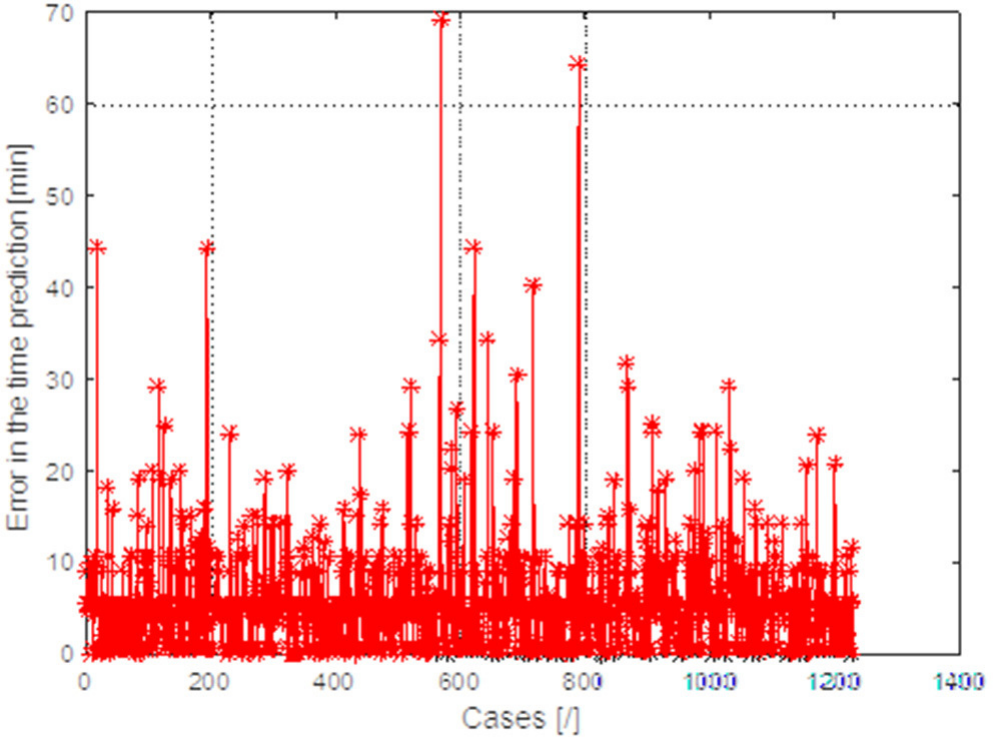
Graph of the maximum absolute error between the predicted surgery time and the actual value obtained for 1,229 different patients obtained for 1,561 different combinations of features.

As mentioned before, the surgery time feature is important to estimate how much time will be employed to perform the procedure. Therefore, an analysis was performed to determine the analytical form of the relationship between these 21 parameters and surgery time. For this purpose, neural networks were used [FF] with backward error propagation with 21 input neurons and 10 neurons in the hidden layer. The maximum absolute error between the predicted surgery time and the actual time was estimated for each trained neural network, for the test vector (2/3 of patients—training vector and 1/3—test vector). The results obtained are shown in Figure 3. Each point on the x axis is a different configuration of features.

For each configuration, the input characteristics change maximum absolute error between the predicted surgery time. The best results were obtained for feature configuration: IOL power + AL + kind of surgeon.

The obtained estimation results and actual measurements of the surgery time for 1,229 patients are shown in Figure 3. The maximum differences in predicting surgery time on average do not exceed 6 min, which is fully acceptable to ophthalmologists.

To sum up, a method for predicting surgery time was developed based on three input features (IOL power + AL + kind of surgeon) with a mean accuracy of 68.4%. Overall mean time of surgery in our study population was 17.58 ± 9.42 min whereas the predicted mean time of surgery according to our model was 17.38 ± 5.19 min, with no clinical or statistical (*p* = 0.39) differences.

## Discussion

Today, cataract surgery is one of the most frequent procedures executed worldwide. It is easy to imagine that it will increase in future, due to the rise of the mean age of the overall population (26). It is a typically highly successful procedure, rates of intra-operative complications have been recently reported to range between 3.8 and 8.4% internationally (5, 6, 10–14).

The pursuit in providing continuously better results after this kind of surgery, also due to higher patient expectations, push physicians to reduce the incidence of complications (26). For this reason, identifying and stratifying patient preoperative risk factors is a topic of increasing interest (4–14).

Previously published studies provide different results compared to those obtained in this case series.

One of the most evaluated strategies to assess complication risks is that proposed by Muhtaseb et al. that considered the most common complications and risk factors reported previously (8). This method has been successfully adopted by the Auckland Cataract Study (5–10) and modified by the same group in 2019 (4, 27).

Nderitu et al. (28), published an interesting article about this topic in a very large series of cases collected in different UK hospitals, evaluating the application of the risk factors previously identified by the UK Cataract National Database.

Different reasons could explain the different results obtained, mainly, this is the first study applying AI analysis to this specific topic. Another one could be the amount of variables collected and evaluated. Indeed, compared to previous studies a completely different analysis strategy has been applied. The present study takes into account a larger number of parameters: the systemic disease, axial length, keratometry, power of the lens planned to implant, eye, kind of surgeon, best corrected visual acuity, refractive defect (not evaluating only the higher ones), and endothelial cell count.

McKay et al. (10), recently published one of the first attempts to identify risk factors and to build a model to predict surgical time. They evaluated several factors: systemic, ocular and surgeon-related, in a single academic center, with bivariate and multivariate regression strategies. In their analysis, they considered several parameters including both ocular and systemic factors, observing that the 10 cases with complications, out of the 1,349 evaluated, were correlated with multiple factors such as male sex, increased body mass index, first-eye surgery, left operative eye, advanced cataract, use of iris hooks, use of Malyugin ring, use of trypan blue, history of diabetic retinopathy, short axial length, and shallow anterior chamber depth. The different results obtained compared to this study could be related to more than one factor: firstly, in our study a different analysis approach, such as AI, has been adopted; then McKay et al. (10) associated longer surgical time with the above mentioned features and, in particular, with resident cases. Longer surgery time, however, does not necessarily mean that a complication occurred; moreover, the data have been collected in 2014 and the devices used are not mentioned, in our study the same phacoemulsification machine was adopted, avoiding bias in the analysis since different machines may not provide the same comfort for all surgeons. Finally, the authors included PEX and previous vitreoretinal surgery in their method to predict surgery time even if they were not connected to complications insurgence.

In this study, a totally different approach strategy has been utilized in order to understand the influence of several ocular and general characteristics on the complication rate.

The multivariate correlation analysis method has a reliability problem caused by the comparison between groups that are very different numerically such as those observed in the analysis of a large amount of cataract surgeries (29, 30) and the set containing the minority that presented complications or when analyzing the overall surgical volume compared to surgery performed by residents. Despite the known limitations (31, 32), artificial intelligence methods have been applied widely in cases like these where classifications of non-analytical characteristics could better adapt (33, 34).

One of the main advantages of this approach is that a customized model for each eye unit can be built to prevent complications and to predict surgery time. One of the major limitations of the previously published study, obviously related to the model used, is that it surely provided advantages in the environment where it was developed but it may be less promising in an environment with different characteristics. It is obvious that an eye with a very short ACD will always be more difficult to treat but the incidence of problems like PEX, BPH, glaucoma, etc., can vary considerably in different countries or regions, thus their influence on the specific population could be under or over estimated by a regression model built in another environment. This problem still remains, in our opinion, using a model built on multicenter data collections, because it cannot account for the differences among risk factor prevalence rates in specific zones.

One of the limitations of this study is that the analysis involved only one ophthalmology unit in one hospital but thanks to this, we built a very specific model for this setting. It would be very interesting to have further studies facing this topic with a similar approach, in order to evaluate the different results analyzing different samples coming from different parts of the world.

Using a similar approach, other units could build their own model and obtain their “specific” risk factors that could be different compared to the ones that could be observed in a very large multi-center study. Moreover, this approach also allows to identify the specific risk for any kind of complication analyzed.

The kind of algorithm purposed in this study can provide a customized prediction of the time of surgery based on the specific characteristics analyzed. This is very important because ocular operating theaters are quite often stressed by a large number of cases and an eventual complication, with the time needed to manage it, causes discomfort to both operators and patients. A better organized time schedule could also provide less stressful conditions for surgeons, providing better outcomes, especially in the case of surgeons in training.

The results observed in this study suggest that the features most frequently involved in cataract complications are the kind of surgeon, axial length and IOL power. It was easy to imagine that operators in training were more strongly connected with complication rates, because of a lack of expertise, but it is interesting to note that the values of AL and IOL power involved were very wide, not related to specific range such as the shorter or longer eyes. The absence of correlations between complications and shorter AL eyes, observed in other studies, could be explained considering the different kind of analysis or also taking in consideration the preoperative organization of our Unit where patient charts are managed directly by residents and further supervised by a trained surgeon while nurses and technicians play a minor role in detailing patient charts. This results in major attention to the particular features of eyes undergoing surgery, sometimes characterized by one or more risk factors. This type of approach can greatly reduce the incidence of complications.

Otherwise, the explanation for the correlation between a wide range of axial length values and complication insurgence could be explained by the underestimation of minor risk factors or a less careful approach when a routine case is being operated. Thus, the key factor to improve the quality of surgical outcome could be to ensure that the best effort is made in all cases, even in those that apparently seem relatively simple. Although this theory could be very hard to validate, it seems to be a very probable scenario.

Surgery timetables are very important as well: last cases of a crowded schedule could induce more stress to the surgeons and, consequently, more errors could occur. In our opinion, surgery timetables should be optimized considering the many different variables involved that can change in the same unit too, week to week. It is important, to have the instruments that can aid in optimizing surgical practice as much as possible also in a customizable way, because every clinical scenario is different and even the same one can change over time. Further studies on this topic should always analyze surgical time aiming to provide more accurate information.

In conclusion, according to the data observed in this study, AI is an interesting tool to build promising customized models to detect risk factors that could influence the insurgence of complications during cataract surgery and to predict surgery time in consideration of the particular features of each patient. Moreover, the data observed in this study highlight the centrality of the eye doctor in carefully evaluating first the patient and then the eyes undergoing surgery. Technology undoubtedly provides improvements to our daily life and to our practice but it should be kept in mind that there is no fail proof solution to prevent complications in surgery and that physicians must never underestimate the most important devices involved: their brains and their abilities.

## Data Availability Statement

The raw data supporting the conclusions of this article will be made available by the authors, without undue reservation.

## Ethics Statement

The studies involving human participants were reviewed and approved by Comitato Etico Università degli Studi della Campania Luigi Vanvitelli - Azienda Ospedaliera Universitaria Luigi Vanvitelli - AORN Ospedali dei Colli. The patients/participants provided their written informed consent to participate in this study.

## Author Contributions

ML, FS, and SS were responsible for the initial plan and study design. RB, AT, and AR collected and extracted data. AR, RK, and KK were responsible for statistical analysis. ML, RB, AT, and KK interpreted data and drafted the manuscript. FS, ML, SS, and RK did a critical revision of the manuscript. All authors contributed to the article and approved the submitted version.

## Conflict of Interest

The authors declare that the research was conducted in the absence of any commercial or financial relationships that could be construed as a potential conflict of interest.
